# Elevated estimated pulse wave velocity and the risk of type 2 diabetes in non-obese young adults: a longitudinal cohort study

**DOI:** 10.1186/s12902-025-01967-4

**Published:** 2025-06-06

**Authors:** Chunxia Zhang, Li Chen, Ri Liu

**Affiliations:** 1 Department of Cardiology, Ningbo Medical Center LiHuiLi Hospital (The Affiliated LiHuiLi Hospital of Ningbo University), Ningbo, Zhejiang China; 2 Department of Interventional Radiology, Ningbo Medical Center LiHuiLi Hospital (The Affiliated LiHuiLi Hospital of Ningbo University), Ningbo, Zhejiang China

**Keywords:** Pulse wave velocity, Type 2 diabetes, Non-obese young adults, Arterial stiffness, Longitudinal study

## Abstract

**Background:**

Arterial stiffness (AS), measured by estimated pulse wave velocity (ePWV), is associated with a higher risk of cardiovascular diseases and type 2 diabetes mellitus (T2DM) in older and obese individuals. However, the role of AS as an early predictor of T2DM in non-obese, young adults remains underexplored. Identifying alternative predictors like AS is crucial for detecting diabetes onset in non-obese and younger populations who may not exhibit traditional risk factors such as high body mass index (BMI).

**Methods:**

A cohort of 9,543 non-obese participants aged 18–49 years from the NAGALA dataset was followed over a median period of 6.3 years. Cox proportional hazard models were used to assess the association between ePWV and T2DM risk, adjusting for multiple covariates, including age, sex, BMI, alcohol consumption, smoking status, and metabolic markers. Subgroup analyses were conducted to evaluate the stability of the association across different groups. Additionally, ROC curve analysis was performed to assess the predictive power of ePWV in T2DM risk.

**Results:**

A total of 110 participants developed T2DM during follow-up. Elevated ePWV was associated with increased T2DM risk (HR 1.36, 95% CI: 1.05–1.75, *P* = 0.018), even after adjusting for multiple covariates. The ROC analysis demonstrated that the inclusion of ePWV in the predictive model (sex + BMI + diastolic blood pressure (DBP) + ePWV) improved the predictive power for T2DM risk, with AUC values increasing in comparison to the model using sex, BMI and DBP alone (10-year AUC: 0.734 vs. 0.679, *P* = 0.016). Subgroup analyses showed that the association between ePWV and T2DM risk was consistent across sex, age, alcohol consumption, and smoking status.

**Conclusions:**

Elevated ePWV independently correlates with a higher risk of T2DM in non-obese young adults.

**Lay summary:**

This study investigates the relationship between elevated arterial stiffness (AS), measured by estimated pulse wave velocity (ePWV), and the risk of developing type 2 diabetes mellitus (T2DM) in non-obese young adults. While AS has been linked to higher T2DM risk in older or obese individuals, this study uniquely focuses on non-obese young adults, a group not typically associated with high diabetes risk. By analyzing data from over 9,500 participants, the research found that even in individuals with a normal body mass index (BMI), higher ePWV is significantly associated with an increased risk of T2DM. This suggests that measuring ePWV could help detect early diabetes risk in people who may not exhibit traditional risk factors, such as high BMI. The findings highlight the importance of vascular health in prevention of diabetes and propose ePWV as a potential tool for early detection in clinical practice.

## Background

Type 2 diabetes mellitus (T2DM) is a growing global health issue, increasingly affecting younger and non-obese populations. Traditionally, the primary risk factors for T2DM have included obesity, age, and family history [1, 2]. However, recent studies have suggested that individuals without obesity are not immune to the risk of developing T2DM, highlighting the need to investigate alternative early indicators [3–6]. Arterial stiffness (AS), a condition characterized by the thickening and stiffening of arterial walls, has been identified as an early indicator of cardiovascular diseases [7], and recent evidence suggests that it may also be associated with the pathogenesis of T2DM [8]. Estimated pulse wave velocity (ePWV) offers a practical and cost-effective alternative to traditional AS measurements like pulse wave velocity (PWV) and ambulatory arterial stiffness index (AASI), which require specialized equipment and are less feasible in routine clinical settings [9]. Calculated from age and mean blood pressure (MBP), ePWV reliably reflects AS and has predictive value for cardiovascular risk, making it an accessible tool for broader clinical use [7]. Understanding the relationship between ePWV and T2DM in a younger, non-obese population is critical, as early detection could offer new pathways for preventive interventions.

In light of the increasing prevalence of T2DM in non-obese populations, there is a pressing need to shift the focus of diabetes prevention strategies towards early detection and novel risk factors. Traditional risk factors, such as obesity and age, may no longer be sufficient for effective risk stratification, especially as evolving lifestyles and dietary habits contribute to shifts in metabolic risk profiles [2]. Emerging markers, like ePWV, provide valuable insights into cardiovascular and metabolic health, particularly in younger, non-obese adults. This study explores the association between elevated ePWV and the incidence of T2DM in non-obese young adults.

## Methods

### Data source

This study utilized data from the NAGALA cohort, a well-established longitudinal dataset from Murakami Memorial Hospital in Japan [10]. The NAGALA database provides extensive health and metabolic data from adult participants, making it suitable for analyzing the development of metabolic disorders such as T2DM. All participants included in this analysis were free from T2DM at baseline, as their FPG levels were below the threshold for diabetes diagnosis. The data are accessible via the DRYAD database (https://datadryad.org/stash/dataset/doi:10.5061/dryad.8q0p192), for secondary analysis, respecting the rights of the original researchers.

### Study population

**Fig. 1:**
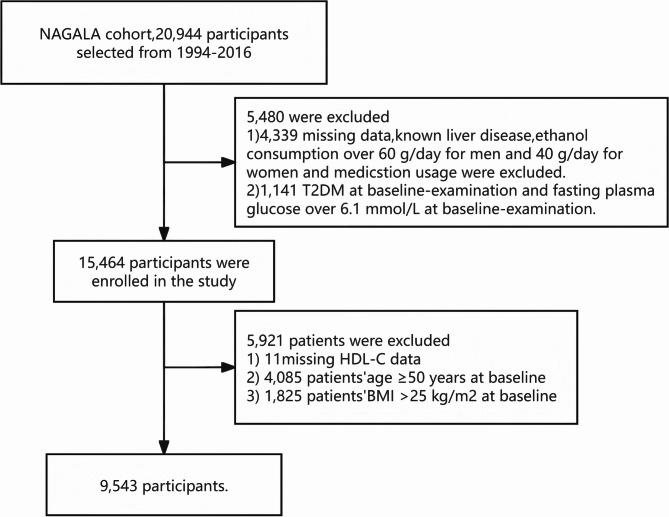
Flowchart of participant selection in the NAGALA Study. Legend: This flowchart illustrates the selection process of participants for the study from the NAGALA cohort, originally consisting of 20,944 individuals enrolled from 1994 to 2016. A total of 5,480 participants were excluded due to missing data, known liver disease, excessive alcohol consumption (over 60 g/day for men and 40 g/day for women), medication usage, existing T2DM at baseline, or fasting plasma glucose over 6.1 mmol/L at baseline. The remaining 15,464 participants were further filtered, excluding 5,921 individuals due to missing HDL-C data, age ≥ 50 years, or BMI ≥ 25 kg/m². This resulted in a final study cohort of 9,543 non-obese participants aged 18–49 years

The initial population for this study comprised 20,944 participants from the NAGALA cohort. A total of 5,480 participants were excluded due to incomplete data, such as missing measurements of High-Density Lipoprotein Cholesterol (HDL-C), or the presence of conditions like liver disease or excessive alcohol consumption. The remaining 15,464 participants were further filtered to focus on non-obese young adults. Participants aged 50 years or older (*n* = 4,085) and those with a body mass index (BMI) over 25 kg/m² [11] (*n* = 1,825) were excluded. After these exclusions, the final study population consisted of 9,543 non-obese participants aged between 18 and 49 years (Fig. 1). The study population was categorized into young adults (18–39 years) and middle-aged adults (40–49 years).

### Data collection and measurements

Data were collected during routine health check-ups, encompassing demographics (age, sex), lifestyle factors (smoking status, alcohol consumption, exercise habits), and clinical measurements. The medical history and lifestyle information were gathered using a standardized self-administered questionnaire, which included questions about smoking and alcohol habits as well as physical activity. Alcohol consumption was categorized based on the type and amount consumed per week in the preceding month, with groups including no or minimal alcohol consumption (< 40 g/week), light (40–140 g/week), moderate (140–280 g/week), and heavy (> 280 g/week). Smoking status was categorized as never, ex-smoker, or current smoker. Regular exercisers were defined as individuals who engaged in any type of sports > 1 time per week.

Key clinical parameters included BMI, systolic blood pressure (SBP), diastolic blood pressure (DBP), pulse pressure (PP), fasting plasma glucose (FPG), triglycerides (TG), total cholesterol (TC), and HDL-C. BMI was determined by dividing weight in kilograms by height in meters squared. PP was determined by subtracting DBP from SBP. Liver function markers such as alanine aminotransferase (ALT), aspartate aminotransferase (AST), and gamma-glutamyl transferase (GGT) were also assessed. All laboratory tests were performed after an overnight fast.

ePWV [7] was calculated using the following formula:

$$\begin{gathered}\:ePWV = 9.587 - 0.402 \times \:age + 4.560 \times \:{10^{ - 3}} \times \:ag{e^2} \hfill \\\,\,\,\,\,\,\,\,\,\,\,\,\,\,\,\, - 2.621 \times \:{10^{ - 5}} \times \:ag{e^2} \times \:MBP \hfill \\\,\,\,\,\,\,\,\,\,\,\,\,\,\,\, + 3.176 \times \:{10^{ - 3}} \times \:age \times \:MBP \hfill \\\,\,\,\,\,\,\,\,\,\,\,\,\,\, - 1.832 \times \:{10^{ - 2}} \times \:MBP, \hfill \\ \end{gathered} $$

where mean blood pressure (MBP) was calculated as follows:

$$\:MBP=DBP+0.4\times\:(SBP-DBP).$$

### Definition of T2DM

Incident T2DM was diagnosed during the follow-up period based on fasting plasma glucose (≥ 7 mmol/L) or HbA1c levels (≥ 6.5%), in accordance with the American Diabetes Association guidelines [12]. Additionally, participants with self-reported physician-diagnosed diabetes were included in the incident T2DM classification [13].

### Statistical analysis

Data were processed and analyzed using Free Statistics software version 1.9, which incorporates the R statistical software version 4.3.2 (https://www.R-project.org, R Foundation). Continuous variables with normal distributions were expressed as mean ± standard deviation (SD), while those with skewed distributions were represented as medians with interquartile ranges (IQR). Comparisons between the three ePWV groups were conducted using the t-test for normally distributed variables and the Wilcoxon rank-sum test for skewed data. Categorical variables were expressed as numbers (percentages) and analyzed using the chi-square test.

To assess differences across the three ePWV groups, the Kruskal-Wallis test or one-way ANOVA was applied, depending on the distribution of the variables. The relationship between ePWV and T2DM risk was analyzed using Cox proportional hazard models. Subgroup analyses were conducted to identify potential effect modifiers based on age group (young adults [18–39 years] vs. middle-aged adults [40–49 years]), sex (male vs. female), alcohol consumption (none vs. light/moderate/heavy), and smoking status (never vs. past/current smoker). For the ROC curve analysis, the discriminatory power of ePWV in predicting T2DM risk was assessed by comparing a model using traditional risk factors [14] (sex + BMI + DBP) to a model incorporating ePWV (sex + BMI + DBP + ePWV). The Area Under the Curve (AUC) was calculated for both models across four follow-up periods: 1 year, 3 years, 5 years, and 10 years. AUC values were compared to evaluate the improvement in prediction when ePWV was included. Analyses were deemed statistically significant with a p-value below 0.05.

## Results

### Baseline characteristics of selected participants

A total of 9,543 participants were included in this study. Table 1 presents the baseline characteristics grouped by tertile of ePWV. The mean age of participants was 39.4 ± 5.7 years, and 51.3% were male. During a median follow-up period of 6.3 years, 110 participants (1.2%) developed T2DM. The values for the tertiles of ePWV were: Tertile 1 (T1) ≤ 6.0 m/s, Tertile 2 (T2) 6.0 < ePWV ≤ 6.5 m/s, and Tertile 3 (T3) > 6.5 m/s. Participants in the highest ePWV tertile (T3) were older and exhibited higher values for BMI, SBP, DBP, TG, and FPG. Additionally, T3 included a larger proportion of smokers and alcohol consumers compared to the lower tertiles (T1 and T2).

**Table 1:** Baseline characteristics of selected participants

Variables	Total (*n* = 9543)	ePWV			
		T1 (*n* = 3180)	T2 (*n* = 3180)	T3 (*n* = 3183)	*p* value
Sex, n (%)					< 0.001
Male	4892 (51.3)	2331 (73.3)	1477 (46.4)	1084 (34.1)	
Female	4651 (48.7)	849 (26.7)	1703 (53.6)	2099 (65.9)	
Age(years), Mean ± SD	39.4 ± 5.7	36.7 ± 5.3	38.9 ± 5.3	42.5 ± 4.8	< 0.001
SBP(mmHg), Mean ± SD	110.6 ± 13.2	98.2 ± 6.9	110.2 ± 6.8	123.5 ± 10.5	< 0.001
DBP(mmHg), Mean ± SD	68.8 ± 9.5	59.6 ± 4.9	68.4 ± 4.3	78.3 ± 7.3	< 0.001
PP(mmHg), Mean ± SD	14.9 ± 6.6	38.6 ± 6.0	41.8 ± 5.9	45.2 ± 6.2	< 0.001
Habit of exercise, n (%)					0.611
No	8038 (84.2)	2694 (84.7)	2676 (84.2)	2668 (83.8)	
Yes	1505 (15.8)	486 (15.3)	504 (15.8)	515 (16.2)	
Alcohol consumption, n (%)					< 0.001
None	7547 (79.1)	2801 (88.1)	2550 (80.2)	2196 (69)	
Light/Moderate/Heavy	1996 (20.9)	379 (11.9)	630 (19.8)	987 (31)	
Smoking status, n (%)					< 0.001
Never	6014 (63.0)	2296 (72.2)	1956 (61.5)	1762 (55.4)	
Past/Current	3529 (37.0)	884 (27.8)	1224 (38.5)	1421 (44.6)	
BMI(kg/m^2^), Mean ± SD	21.0 ± 2.1	20.0 ± 2.0	21.1 ± 2.1	21.9 ± 2.0	< 0.001
HDL-C(mmol/L), Mean ± SD	1.5 ± 0.4	1.6 ± 0.4	1.5 ± 0.4	1.5 ± 0.4	< 0.001
TC(mmol/L), Mean ± SD	4.9 ± 0.8	4.8 ± 0.8	4.9 ± 0.8	5.2 ± 0.8	< 0.001
TG(mmol/L), Mean ± SD	0.8 ± 0.6	0.6 ± 0.3	0.8 ± 0.5	1.0 ± 0.7	< 0.001
HbA1c, (mmol/mol), Mean ± SD	32.4 ± 3.3	32.2 ± 3.2	32.4 ± 3.3	32.6 ± 3.4	< 0.001
FPG(mmol/L), Mean ± SD	5.1 ± 0.4	4.9 ± 0.4	5.1 ± 0.4	5.2 ± 0.4	< 0.001
ePWV(m/s), Mean ± SD	6.6 ± 0.8	5.8 ± 0.3	6.5 ± 0.2	7.5 ± 0.6	< 0.001
Follow-up(years), Mean ± SD	6.3 ± 3.8	6.1 ± 3.8	6.4 ± 3.8	6.5 ± 3.9	< 0.001
T2DM, n(%)					< 0.001
No	9433 (98.8)	3162 (99.4)	3154 (99.2)	3117 (97.9)	
Yes	110 ( 1.2)	18 (0.6)	26 (0.8)	66 (2.1)	
ALT(IU/L), Median (IQR)	15.0 (12.0, 21.0)	13.0 (11.0, 17.0)	15.0 (12.0, 21.0)	17.0 (13.0, 24.0)	< 0.001
AST(IU/L), Median (IQR)	16.0 (13.0, 20.0)	15.0 (13.0, 18.2)	16.0 (13.0, 20.0)	17.0 (14.0, 21.0)	< 0.001
GGT(IU/L), Median (IQR)	14.0 (11.0, 19.0)	12.0 (10.0, 15.0)	14.0 (11.0, 19.0)	17.0 (12.0, 25.0)	< 0.001

Note: Data presented are mean ± SD, median (Q1–Q4), or n (%); T1, T2, T3 are tertile of ePWV. Abbreviations: BMI, body mass index; ALT, alanine aminotransferase; AST, aspartate aminotransferase; gamma-glutamyl transferase; SBP, systolic blood pressure; DBP, diastolic blood pressure; pulse pressure, PP; HbA1c, hemoglobin A1c; FPG, fasting plasma glucose; HDL-C, high-density lipoprotein cholesterol; ePWV, estimated pulse wave velocity; T2DM, Type 2 Diabetes

### Cox proportional hazard model

The Cox proportional hazard models revealed a significant association between elevated ePWV and increased risk of T2DM (Table 2). As a continuous variable, each 1 m/s increase in ePWV was associated with an 85% higher risk of developing T2DM in the unadjusted model (HR: 1.85, 95% CI: 1.53–2.23, *P* < 0.001) (Table 2).

**Table 2:** Associations between ePWV and T2DM in the multiple COX regression model

Variable	Non-adjusted Model		Model 1		Model 2		Model 3	
	HR(95%) CI	*P*-value	HR(95%) CI	*P*-value	HR(95%) CI	*P*-value	HR(95%) CI	*P*-value
ePWV, m/s	1.85 (1.53 ~ 2.23)	< 0.001	1.56(1.12 ~ 2.16)	0.008	1.68 (1.21 ~ 2.33)	0.002	1.58 (1.14 ~ 2.2)	0.007

Notes: Data presented are HRs and 95% CIs. Model I: adjusted for age, sex, BMI, PP. Model II: adjusted for model1 + Alcohol consumption, Habit of exercise, Smoking status. Model III: adjusted for model2 + ALT, AST, GGT, HDL-C, TC, TG. Abbreviations: ePWV, estimated pulse wave velocity; BMI, body mass index; pulse pressure, PP; ALT, alanine aminotransferase; AST, aspartate aminotransferase; GGT, gamma-glutamyl transferase; HDL-C, high-density lipoprotein cholesterol; TC, total cholesterol; TG, triglyceride

After adjusting for age, sex, BMI and PP in Model 1, the association remained significant but was attenuated (HR 1.56, 95% CI: 1.12–2.16, *P* = 0.008). With additional adjustments for alcohol consumption, exercise, and smoking status in Model 2, the HR increased slightly to 1.68 (95% CI: 1.21–2.33, *P* = 0.002). In the fully adjusted Model 3, which included liver enzymes (ALT, AST, GGT) and lipid profiles (HDL-C, TC, TG), the association remained significant, with an HR of 1.58 (95% CI: 1.14–2.2, *P* = 0.007).

### Threshold effect analysis of ePWV on incident T2DM

**Fig. 2:**
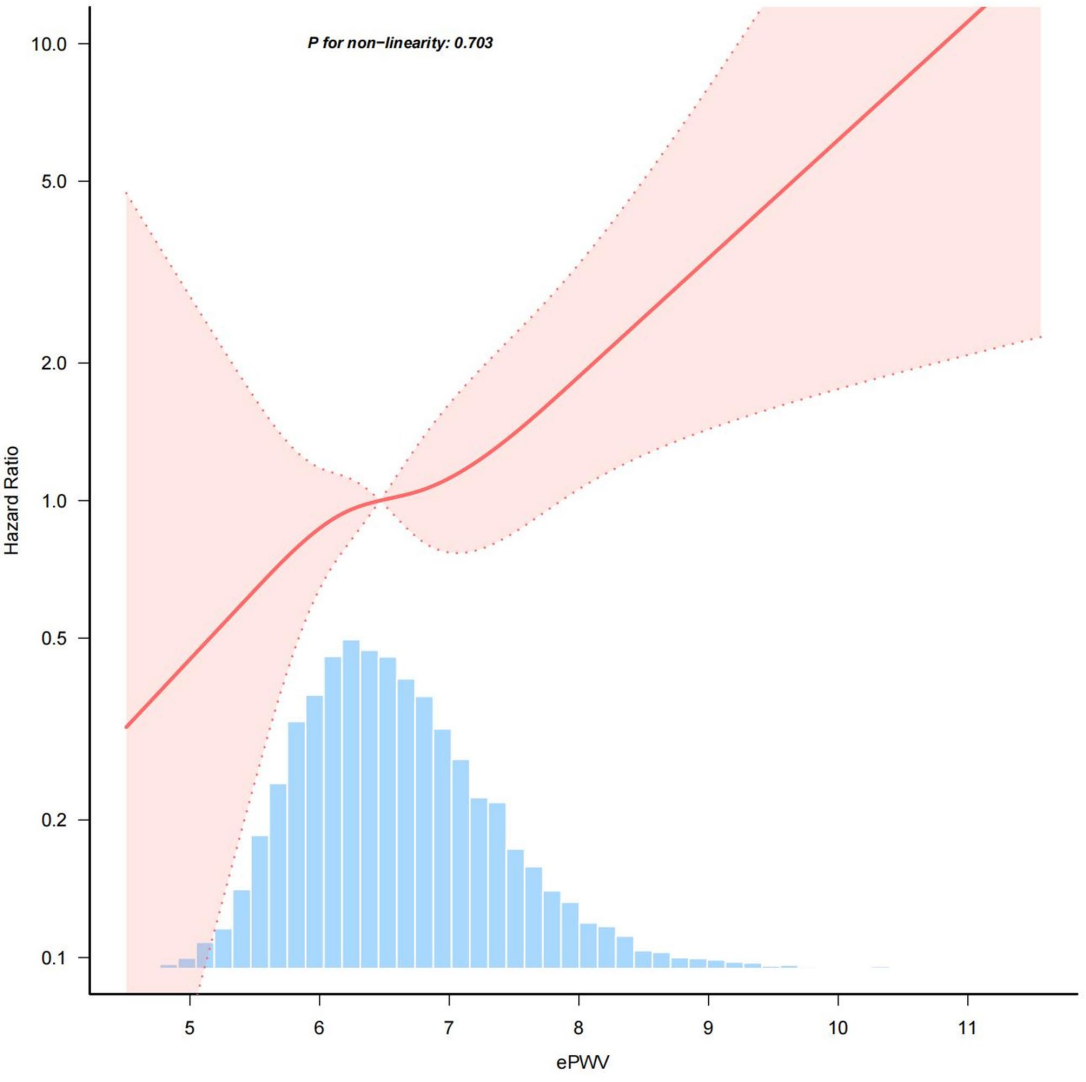
Association between ePWV and risk of T2DM incidence. Legend: This figure illustrates the relationship between estimated pulse wave velocity (ePWV) and the hazard ratio (HR) for the risk of T2DM incidence. The red line represents the HR curve, while the shaded area indicates the 95% confidence interval. The histogram at the bottom shows the distribution of ePWV in the study population. No significant non-linearity was observed (*P* = 0.971)

A Cox proportional hazards regression model with cubic spline functions was employed to investigate the relationship between ePWV and T2DM incidence (Fig. 2). After adjusting for potential confounders, including age, sex, BMI, PP, alcohol consumption, exercise habits, smoking status, liver enzymes (ALT, AST, GGT), and lipid profile (HDL-C, TC, TG), the analysis revealed a positive linear relationship between ePWV and the risk of T2DM.

### Subgroup analysis

**Fig. 3:**
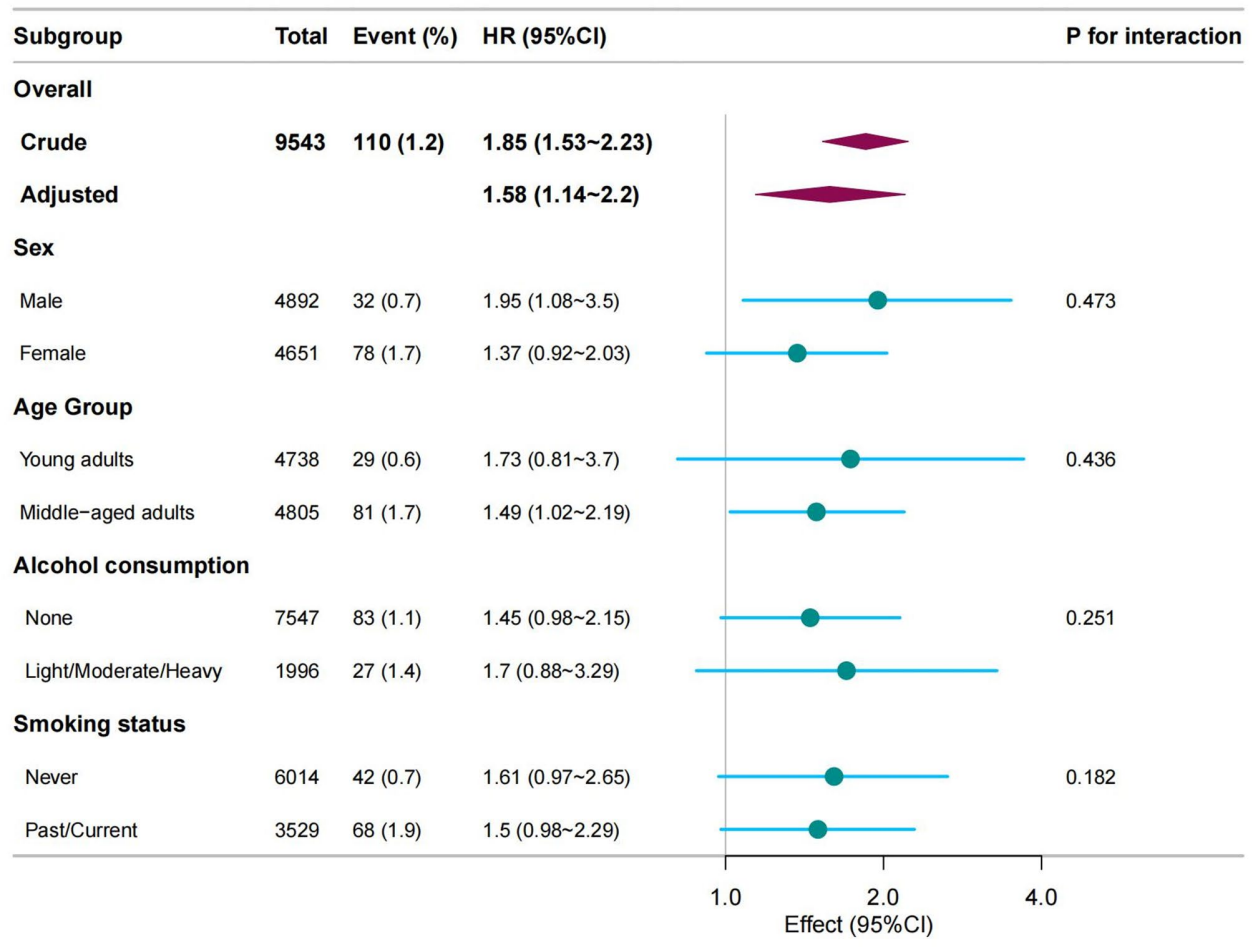
Forest plot of hazard ratios (HRs) for the association between ePWV and T2DM risk across subgroups. Legend: This forest plot presents the hazard ratios (HRs) for the association between estimated pulse wave velocity (ePWV) and the risk of T2DM in various subgroups. The overall adjusted HR was 1.36 (95% CI: 1.05–1.75). Subgroup analyses were performed by sex, age, alcohol consumption, and smoking status, showing no significant interaction effects. The HRs for each subgroup are displayed with 95% confidence intervals, with p-values for interaction indicating no statistically significant differences across subgroups

A subgroup analysis was conducted to examine the relationship between ePWV and T2DM risk across different groups (Fig. 3). For each subgroup, the analyses were adjusted using the same covariates as in Model 3.

As observed in the forest plot, the association between ePWV and T2DM risk was stronger in male participants (HR: 1.95, 95% CI: 1.08–3.5) compared to females (HR: 1.37, 95% CI: 0.92–2.03). Additionally, a slightly higher risk was observed in young adults (HR: 1.73, 95% CI: 0.81–3.7) compared to middle-aged adults (HR: 1.49, 95% CI: 1.02–2.19), although no significant interaction was found between age and ePWV.

The association between ePWV and T2DM risk was not significantly modified by alcohol consumption (HR: 1.45, 95% CI: 0.98–2.15 for none vs. 1.7, 95% CI: 0.88–3.29 for light/moderate/heavy consumption), nor by smoking status (HR: 1.61, 95% CI: 0.97–2.65 for never smokers vs. 1.5, 95% CI: 0.98–2.29 for past/current smokers).

### ROC analysis of ePWV’s contribution to T2DM risk prediction

**Fig. 4:**
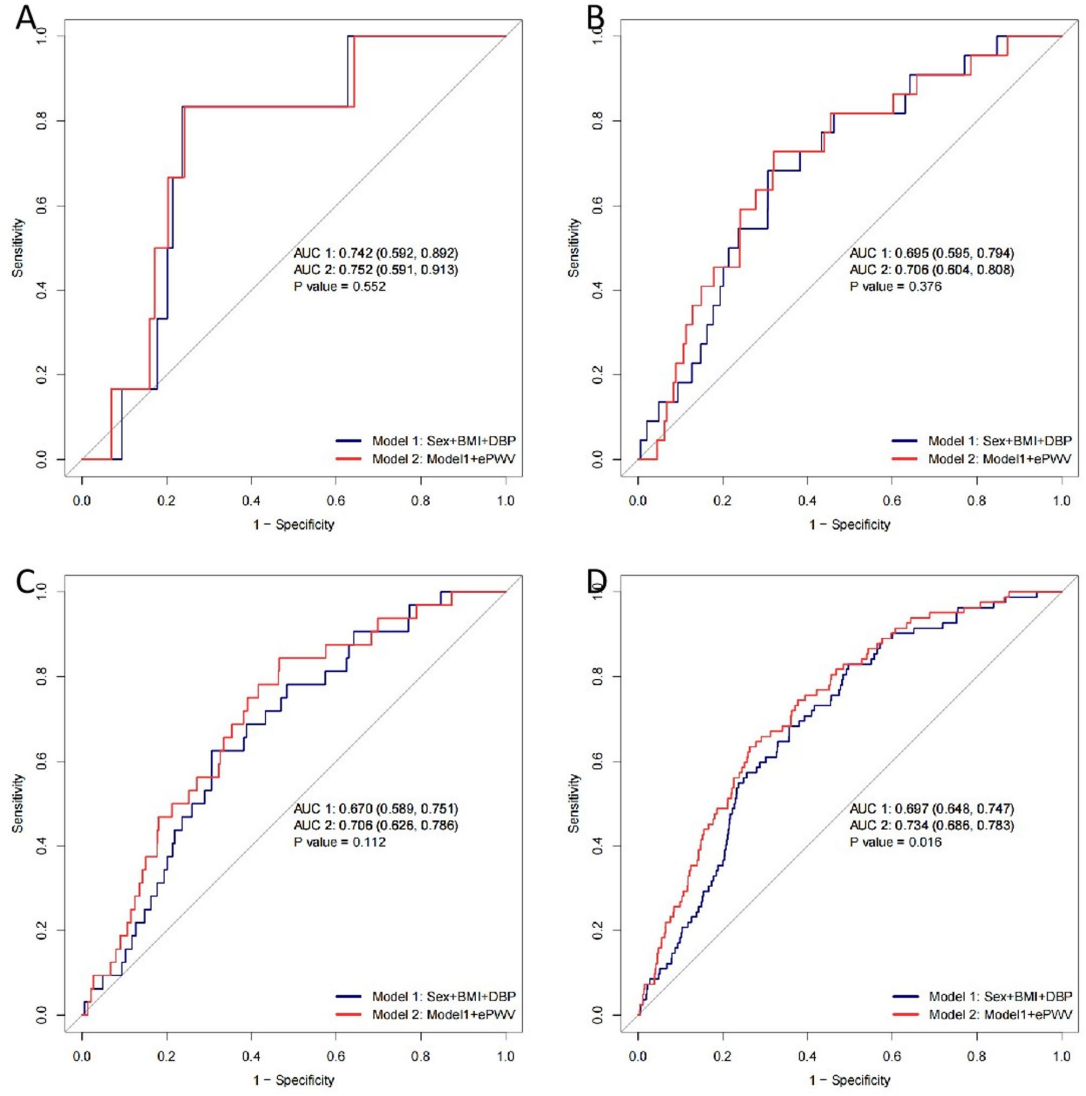
Receiver operating characteristic (ROC) Curve Analysis of ePWV in Predicting T2DM Risk. Legend: ROC curves comparing the diagnostic performance of Model 1 (Sex + BMI + DBP) and Model 2 (Model 1 + ePWV) for predicting type 2 diabetes mellitus (T2DM) at different follow-up periods: **(A)** 1-year, **(B)** 3-year, **(C)** 5-year, and **(D)** 10-year incidence. The area under the curve (AUC) values and corresponding 95% confidence intervals are presented for each model. Across different follow-up durations, Model 2 (red line, with ePWV) consistently showed higher AUC values than Model 1 (blue line, without ePWV). Notably, in **(D)** the 10-year follow-up, Model 2 demonstrated a improved diagnostic ability compared to Model 1 (AUC: 0.734 vs. 0.697, *P* = 0.016)

The analysis was conducted for four follow-up periods: 1 year, 3 years, 5 years, and 10 years(Fig. 4. A, B, C, D). As shown in Fig. 4, the AUC values for Model 1(sex + BMI + DBP) were 0.742, 0.695, 0.670, and 0.697 for the respective follow-up periods of 1 year, 3 years, 5 years, and 10 years. When ePWV was added to the model (Model 1 + ePWV), the AUC values increased to 0.752, 0.705, 0.705, and 0.734 for the same follow-up periods.Notably, while the AUC improvements were not statistically significant for the 1-year, 3-year, and 5-year follow-ups (*P* = 0.552, 0.376, 0.112, respectively), the inclusion of ePWV significantly improved the predictive accuracy of the model for the 10-year follow-up (*P* = 0.016, Fig. 4D).

## Discussion

This retrospective cohort study of a Japanese population found a positive linear relationship between elevated ePWV and the incidence of T2DM in non-obese young adults. This association remained significant after adjusting for key covariates. Notably, the relationship between ePWV and T2DM risk was consistent across subgroups, including sex, age, alcohol consumption, and smoking status. Additionally, adding ePWV to the model (sex + BMI + DBP) improved the predictive accuracy for T2DM risk in the 10-year follow-up (Fig. 4D). Although the AUC increase was modest, this should be interpreted in the context of our study population. Non-obese young adults are typically not considered high-risk for T2DM and may be under-screened in clinical settings [15]. Therefore, the ability of ePWV to improve discrimination, even marginally, suggests its utility in uncovering early vascular changes that might otherwise go undetected by conventional indicators like BMI or age.

Diabetes is a chronic metabolic disease that can cause multi-organ dysfunction and failure, significantly impacting health. Thus, early screening and prevention are essential [2, 16, 17]. AS, a risk factor for hypertension, cardiovascular disease, chronic kidney disease, and cognitive decline, has also been identified as a potential risk factor for diabetes [18, 19]. Our findings align with previous studies that have demonstrated a connection between AS and the risk of T2DM [20–23]. For example, Muhammad et al. reported an association between increased AS measured by PWV and the incidence of T2DM in a broader population [24]. A study conducted by Mengyi Zheng et al. in 2020 in China evaluated the relationship between diabetes and brachial-ankle PWV (baPWV), identifying AS as an independent risk factor for diabetes [18]. Similarly, a Swedish study involving 3,734 individuals found that the relative risk of developing diabetes in the highest carotid-femoral PWV (cfPWV) tertile was 3.24 times higher than that in the lowest tertile [8]. The Candesartan Anti-hypertensive Survival Assessment in Japan (CASE-J) trial showed that for every standard deviation increase in PP, the risk of developing diabetes increased by 44%, identifying peripheral PP as an independent predictor of new-onset diabetes in high-risk Japanese hypertensive patients [24]. Compared to other AS indicators, ePWV can be derived from routine clinical data such as age and blood pressure, making it a more accessible and cost-effective tool for assessing cardiovascular health in large epidemiological studies. Importantly, ePWV has been shown to correlate strongly with carotid-femoral PWV (cfPWV), the gold standard for measuring AS [25]. This close correlation, along with ePWV’s ease of calculation, enhances the feasibility of identifying individuals at higher risk for T2DM, especially in non-obese populations, across various healthcare settings. Further studies are needed to explore its potential for inclusion in predictive models for T2DM risk.

The potential mechanisms by which AS led to T2DM are as follows. First, PP elevation due to AS may lead to endothelial dysfunction [26]. Endothelial dysfunction and impaired endothelium-dependent vasodilation may exacerbate insulin resistance by impairing glucose delivery to key target tissues, such as the pancreas, liver, and muscles, which precedes the development of diabetes [27]. Second, AS may affect the microvascular health of the pancreas, potentially leading to endocrine dysfunction. Microvascular dysfunction and skeletal muscle remodeling may contribute to insulin resistance [28]. The microvascular changes associated with increased AS may impair insulin-mediated muscle perfusion and alter glucose metabolism [29]. Mendelian randomization studies have shown that genetically determined reduced insulin secretion is linked to AS [30, 31]. Finally, chronic low-grade inflammation and increased oxidative stress may be common risk factors for both diabetes and AS [32].

However, several limitations should be noted. As this study is based on a secondary analysis of publicly available NAGALA cohort data, detailed information on blood pressure measurement methods was not available. Although ePWV is a feasible surrogate marker for AS, it may not fully capture the nuances of direct PWV measurements obtained through more specialized techniques like cfPWV [20]. Additionally, our study lacked data on diabetes medication use, which may lead to a potential underestimation of diabetes incidence. We have now explicitly acknowledged this limitation and emphasized the need for future studies incorporating medication data to improve risk prediction accuracy. Furthermore, our study’s focused on non-obese young adults in Japan, which may limit the generalizability of the findings to other populations with different risk profiles [1]. Lastly, despite controlling for many potential confounders, residual confounding from unmeasured variables, such as dietary factors or genetic predisposition, cannot be completely ruled out.

Future research should focus on using direct AS measurement methods to validate the current findings and expand the study sample to include more diverse ethnic groups. By integrating genetic and dietary data, we can achieve a more comprehensive understanding of the interaction mechanisms between vascular health and diabetes risk. In addition to ePWV, studies should explore other biomarkers, such as inflammatory markers, to further elucidate the mechanisms linking AS and T2DM.

## Conclusions

In conclusion, elevated ePWV was positively and linearly associated with an increased risk of T2DM in a cohort of non-obese young adults in Japan. This relationship remained significant after adjusting for age, sex, BMI, smoking status, alcohol consumption, liver enzymes, and lipid profiles.

## Data Availability

The data used in this study were obtained from the NAGALA (NAfld in Gifu Area, Longitudinal Analysis) cohort, which is publicly available through the DRYAD database. Access to the dataset can be granted through the following link: https://doi.org/10.5061/dryad.8q0p192.

## Abbreviations

AS: Arterial stiffness
BMI: Body Mass Index
PP: Pulse pressure
ePWV: Estimated Pulse Wave Velocity
baPWV: Brachial-ankle PWV
cfPWV: Carotid-femoral PWV
AASI: Ambulatory Arterial Stiffness Index
FPG: Fasting Plasma Glucose
GGT: Gamma-Glutamyl Transferase
HDL-C: High-Density Lipoprotein Cholesterol
HR: Hazard Ratio
SBP: Systolic Blood Pressure
DBP: Diastolic Blood Pressure
MBP: Mean Blood Pressure
T2DM: Type 2 Diabetes Mellitus
ALT: Alanine Aminotransferase
AST: Aspartate Aminotransferase
TC: Total Cholesterol
TG: Triglycerides
TyG: Triglyceride-Glucose
SD: Standard Deviation
IQR: Interquartile Ranges
